# Gastric Antral Vascular Ectasia in Systemic Sclerosis: Current Concepts

**DOI:** 10.1155/2015/762546

**Published:** 2015-11-08

**Authors:** Raphael Hernando Parrado, Hernan Nicolas Lemus, Paola Ximena Coral-Alvarado, Gerardo Quintana López

**Affiliations:** ^1^Universidad de los Andes, Hospital Universitario Fundación Sante Fe de Bogotá, Bogotá, Colombia; ^2^School of Medicine, Universidad de los Andes, Bogota, Colombia; ^3^Department of Rheumatology, Hospital Universitario Fundación Santa fe de Bogotá, Bogota, Colombia; ^4^School of Medicine, Universidad Nacional de Colombia, Bogota, Colombia

## Abstract

*Introduction*. Gastric antral vascular ectasia (GAVE) is a rare entity with unique endoscopic appearance described as “watermelon stomach.” It has been associated with systemic sclerosis but the pathophysiological changes leading to GAVE have not been explained and still remain uncertain. *Methods*. Databases Medline, Scopus, Embase, PubMed, and Cochrane were searched for relevant papers. The main search words were “Gastric antral vascular ectasia,” “Watermelon Stomach,” “GAVE,” “Scleroderma,” and “Systemic Sclerosis.” Fifty-four papers were considered for this review. *Results*. GAVE is a rare entity in the spectrum of manifestations of systemic sclerosis with unknown pathogenesis. Most patients with systemic sclerosis and GAVE present with asymptomatic anemia, iron deficiency anemia, or heavy acute gastrointestinal bleeding. Symptomatic therapy and endoscopic ablation are the first-line of treatment. Surgical approach may be recommended for patients who do not respond to medical or endoscopic therapies. *Conclusion*. GAVE can be properly diagnosed and treated. Early diagnosis is key in the management of GAVE because it makes symptomatic therapies and endoscopic approaches feasible. A high index of suspicion is critical. Future studies and a critical review of the current findings about GAVE are needed to understand the role of this condition in systemic sclerosis.

## 1. Introduction

Gastric antral vascular ectasia (GAVE) is a rare clinical condition related to chronic gastrointestinal bleeding and iron deficiency anemia. It was first described in 1953 by Rider et al. [1] and fully defined by Jabbari et al. in 1984 [2]. GAVE is characterized by a unique endoscopic appearance of rough parallel folds and dilated blood vessels departing from the pylorus and converging in the gastric antrum. This appearance is usually described as “watermelon stomach” [2–6]. GAVE has been associated with diversified medical conditions such as hepatic cirrhosis, chronic renal failure, hypertension, chronic pulmonary disease, and diabetes. It has also been associated with autoimmune diseases, including Raynaud's phenomena, rheumatoid arthritis, polymyalgia rheumatic, primary biliary cirrhosis, and systemic sclerosis (diffuse and limited); still, the pathophysiological changes leading to GAVE have not been fully explained and remain uncertain [7, 8]. This paper gives an overview of the published literature on GAVE in systemic sclerosis with emphasis on pathophysiology and clinical presentation to improve early diagnosis and treatment.

## 2. Materials and Methods

**Figure 1:**
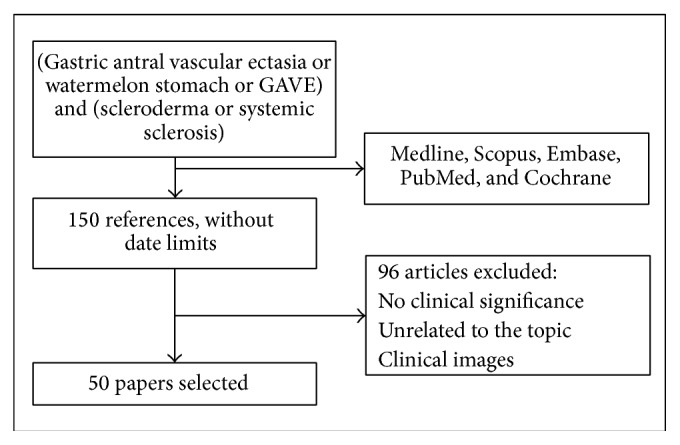
Systemic research and article selection.

The databases Medline, Scopus, Embase, PubMed, and Cochrane were searched in order to retrieve all publications on systemic sclerosis and GAVE as a manifestation of systemic sclerosis. We used MeSH and non-MeSH terms, including “Gastric antral vascular ectasia,” “Watermelon Stomach,” “GAVE,” “Scleroderma,” and “Systemic Sclerosis.” Papers written in English and Spanish were included. There were no limits based on publication date. The search resulted in 135 papers that were assessed by the authors according to whether they contributed evidence to the review topics. Finally, 54 papers were considered to be eligible for inclusion in this review (Figure 1).

## 3. Prevalence and Incidence

GAVE is a rare entity in the spectrum of manifestations of systemic sclerosis, even though it is responsible for persistent chronic gastrointestinal bleeding in these patients [9, 10]. Gastrointestinal manifestations are seen clinically in the majority of patients with systemic sclerosis, and gastric antral vascular ectasia is one of the recognized vascular changes seen in this patient population [11]. A prospective study showed that up to 82% of systemic sclerosis patients have alterations in the esophageal manometry [12, 13]. Additionally, a large retrospective study including 264 patients with limited and diffuse systemic sclerosis reported a prevalence of clinically evident GAVE of 5.7% [14]. However the real prevalence is probably higher as patients consult only when they are symptomatic or when anemia of unknown origin is diagnosed. A recent study (SCOT trial—Scleroderma: Cyclophosphamide Or Transplant study), which included endoscopic evaluation on asymptomatic patients, reported a prevalence of 22.3% [15].

Cohort studies have shown different prevalence of GAVE, which makes it difficult to determine its presence in patients with systemic sclerosis (Table 1). More recently, prevalence studies have reported two characteristics of endoscopic appearances of GAVE with different epidemiological aspects: patients with cirrhosis and diffuse appearance of GAVE and noncirrhotic patients with typical watermelon appearance [16, 17]. Still, the low number of patients in each study limits its power to establish a possible association. The dearth of prevalence trials in the literature reflects the need for more trials to establish how many patients with systemic sclerosis end up with GAVE; differences between countries are needed to determine possible environmental etiology.

In larger case series, GAVE was found to be the cause of severe upper gastrointestinal bleeding in 3.9% of the cases. It is more common in women than men (5 : 1) and the median age of presentation is 70 years [20]. Studies have not confirmed if GAVE is associated more with diffuse systemic sclerosis than with limited systemic sclerosis. The consensus, however, is that patients with diffuse systemic sclerosis develop GAVE earlier than patients with limited systemic sclerosis, although GAVE is more prevalent in the limited type [19].

## 4. Risk Factors

Diverse studies have suggested different medical risk factors that determine the development of GAVE. Risk factors include hypergastrinemia, proliferation of neuroendocrine cells, and alterations in hormone levels of prostaglandin E2 (PGE2), 5-hydroxytryptamine, and vasoactive intestinal polypeptide (VIP) [21, 22]. The last hormones were proposed after the evidence of their presence in the gastric lamina propria. Their action seems to lead to local vasodilation and tendency to bleed [23].

Recent research studies have established that antibodies are an important risk factor in the progression of systemic sclerosis and the apparition of GAVE. Indeed, several types of antibodies, such as anti-RNAP III, have been confirmed as an etiological factor [24]. Additional studies show that systemic sclerosis is an important risk factor to develop GAVE and tilts towards the limited type as GAVE was associated with absence of anti-topoisomerase I antibodies (anti-Scl-70) [19].

## 5. Pathogenesis

The exact cause of GAVE is not known. Some authors propose that it is caused by a loose connection between the distal gastric mucosa and the adjacent muscularis externa. This loosening can cause prolapse of the antral mucosa in the pylorus and development of GAVE [20, 25]. Quintero et al. theorize that strong peristaltic movements can induce prolapse and trauma of antral mucosa and secondary blood vessel obstruction, which can lately lead to fibromuscular hyperplasia and vascular ectasia [21]. However in a large subset of patients with GAVE (between 17% and 57%) the problem is not confined to the antrum but extends into the intestine where a reduction of migratory complexes occurs leading to bacterial overgrowth [26].

Several authors also suggest that GAVE is a vascular manifestation of systemic sclerosis. Up to 60% of GAVE patients will develop skin telangiectasias. Additionally, they have observed histopathologic similarities in skin biopsies of patients with systemic sclerosis and in gastric mucosal biopsies from GAVE patients in terms of capillary dilation, fibrin deposits, and platelet thrombosis [5].

Other authors have linked GAVE with an autoimmune process. Supporting this theory is the fact that GAVE has been associated with other autoimmune diseases in population studies. As we have previously observed, several autoantibodies have been detected in patients with GAVE: antinuclear antibodies (ANAs), anti-centromere and anti-RNA helicase II, especially in patients diagnosed with systemic sclerosis and GAVE [5, 27]. It has been suggested that these antibodies could possibly have a cross-reaction with specific proteins in the gastric mucosa and submucosa that could lead to the clinical findings of this entity [28].

## 6. Clinical Findings and Diagnosis

Although there are reported cases when GAVE is the only manifestation of systemic sclerosis [29], studies have shown that the majority of patients with GAVE, around 60%, have telangiectasias of the skin [30]. Most of the patients present either with symptomatic iron deficiency anemia (weakness, fatigue, or dyspnea) or asymptomatic anemia (with laboratory findings like low hemoglobin and mean corpuscular volume) [19, 31]. The primary physician must suspect GAVE when the anemia is refractory to regular treatment [32]. Therefore an early diagnosis depends on a high index of suspicion.

Patients with diffuse systemic sclerosis can present with occult fecal blood, hematochezia, or hematemesis [14, 25]. One-third can present with asymptomatic rectal bleeding and some patients can even present with abdominal pain or gastric outlet obstruction [33]. In the study done by Marie et al. in 264 patients, the mean of time between the beginning of the disease and the diagnosis is three years with a mean hemoglobin of 8.2 g/dL [14].

Valuable aids in diagnosing GAVE are digestive endoscopy, enteroscopy, red blood cell scan, and video capsule endoscopy. The upper GI endoscopy however is most often used. As we have stated previously anemia, especially if symptomatic and refractory, is an important indication for an endoscopy besides any sign or other symptoms of bleeding or reflux. The objective of the endoscopy is to quantify the extent of mucosal damage in the esophagus and the stomach, detect* Helicobacter pylori* infections, and evaluate the presence of GAVE [34]. As all of these findings are characteristic of early systemic sclerosis, authors recommend doing a complete GI evaluation as soon as the diagnosis is made even when the patient is asymptomatic [34–37].

Additional aids are only recommended when initial treatment fails or when endoscopy is not feasible [38]. In this context, it is important to note that upper GI bleeding in these patients can be also caused by AV malformations, venous ectasia, gastric telangiectasia, hemangioma, and angiosarcoma [39].

## 7. Endoscopic Appearance

The GAVE endoscopic appearance often mimics that of portal hypertensive gastropathy (PHG) or antral gastritis. It is useful to emphasize that GAVE is most commonly limited to the antrum, whereas PHG typically involves the fundus and the corpus of the stomach [20, 25]. The endoscopic appearance of GAVE is classically characterized by red stripes or multiple longitudinal folds from the pylorus through the antrum. Alternatively multiple red dots can be seen. Biopsies normally show capillary dilation with focal intravascular thrombi and muscular hyperplasia of the lamina propria and multiple tortuous submucosal capillaries [2].

## 8. Management

Management of GAVE ranges from symptomatic therapy and noninvasive medical therapy to corrective endoscopic procedures and to surgical interventions. Symptomatic therapy includes iron deficit correction, proton pump inhibitors, and blood transfusion if anemia is very symptomatic and severe [14]. Some patients might need multiple blood transfusions. A recent study in 77 patients with GAVE showed that the mean of units was 4 [9, 14]. Other symptomatic therapies aim to treat coagulopathy and avoid substances that exacerbate the condition: NSAIDs, antiplatelet agents, and other agents such as ginko [14, 20].

There is a case report of a patient treated with cyclophosphamide and methylprednisolone leading to complete and sustained resolution of GAVE in association with systemic sclerosis [40]. Three additional patients with systemic sclerosis and severe GAVE showed remarkable clinical and endoscopic improvement following intravenous cyclophosphamide treatment in a retrospective review of clinical records and endoscopy imaging; authors considered that remission was a result of immunosuppression [41]. Papachristos et al. also published two case reports with significant improvement of refractory GAVE after administration of IV cyclophosphamide [42]. The use of ethinylestradiol and norethisterone with symptomatic and not endoscopic resolution has been reported [43–45]. Several case reports describe successful treatment with histamine antagonists, calcitonin, tranexamic acid, alpha interferon, serotonin antagonists, and thalidomide [46].

Early diagnosis is key in the management of GAVE because it makes an endoscopic approach feasible. The first step towards this goal is to have a high index of suspicion for changes in hemoglobin levels or symptoms of gastrointestinal bleeding. Patients with the localized form of systemic sclerosis have a good prognosis after local therapy, whereas patients with the diffuse form are more difficult to treat. It has been observed that this last subset of patients requires more endoscopic interventions and blood transfusions and they are at greater risk of renal crisis [30].

Different options have been proposed for endoscopic treatment of GAVE. The technique most commonly used is argon plasma coagulation (APC) and is considered one of the best endoscopic therapeutic options [16, 47]. It has been shown to be safe and effective choice with low risk of complications in a cohort of 5 patients with a sustained rise in hemoglobin level and abolished transfusion dependence after treatment [48]; it has also been successful in a patient with recurrent blood loss and transfusion dependent anemia associated with end-stage renal disease [49]. The largest case series of APC treatment reported an efficacy of 90%–100% [48, 50], with no further need for blood transfusions and an increase of hemoglobin level in almost all patients [28]. This technique can cause discomfort as argon gas causes gastric distension [51]. Other complications reported are hemorrhage, perforation, antral stenosis, and sepsis [50, 52]. Scars from APC ablation might lead to deformity of the antrum and recurrence of GAVE [53].

Neodymium-yttrium aluminium garnet (Nd:YAG) laser photocoagulation has also been used to control gastrointestinal bleeding caused by GAVE. In a series of 45 patients treated with Nd:YAG transfusion requirements were abolished in 85% and hemoglobin levels normalized in 87% over a median follow-up period of 2 years with no mayor complications [25]. In another series of 77 patients with systemic sclerosis, 40 patients successfully underwent Nd:YAG [14]. Treatment with Nd:YAG laser photocoagulation therapy has been successful in preventing surgery in patients with anemia secondary to GAVE [54, 55].

Several case reports have reported novel techniques such as band endoscopic ligation [56], endoscopic ablation with forceps [45], and monopolar electrocoagulation and injection of 5% polidocanol [57]. Although results are often successful, prospective studies are needed before providing any definitive conclusion. Bhattacharyya et al. also report a case series with autologous stem cell transplant as an effective disease-modifying therapy for GAVE [58]. This novel approach needs more prospective studies to ascertain its benefit in patients with systemic sclerosis.

The surgical approach includes gastrectomy and antrectomy [59]. They may be the only reliable approach for achieving a cure and eliminating transfusion dependency, but they are rarely used, as endoscopic interventions are widely available. Surgical approach should be reserved for patients who do not respond to medical and/or endoscopic therapies. It must be noted that antrectomy is a procedure with significant morbidity and mortality. A mortality rate of 7.4% was reported in surgical patients with GAVE not linked to systemic sclerosis [60].

Therapeutically outcomes are difficult to assess due to the variety of case reports and the lack of clinical trials published in the literature. As explained above, most of the articles in databases are case reports with several cases reflecting many times empiric treatments and patients with different characteristics such as demographics, time of onset of the disease, and comorbidities. This limits the ability to establish a statistical significance based on intervention effects due to the absence of homogeneity in the intervention and clinical outcome. Randomized clinical trials comparing different types of treatment against a gold standard, with groups sharing clinical characteristics, are needed in the future to perform an adequate statistical analysis.

## 9. Conclusions

Gastric antral vascular ectasia (GAVE) in patients with systemic sclerosis, though a rare and poorly understood condition, can be properly diagnosed and successfully treated. Early diagnosis is key in the management of GAVE because it makes symptomatic therapies and endoscopic approaches feasible. The first step towards this goal is to have a high index of suspicion for changes in hemoglobin levels or symptoms of gastrointestinal bleeding. GAVE may be underdiagnosed because observational studies of screening endoscopies in asymptomatic patients with diffuse systemic sclerosis show that 22.3% of them had silent GAVE. Future studies are needed to determine the prevalence of GAVE in diverse geographical settings, understand the role of GAVE in systemic sclerosis, improve early diagnosis, and determine therapeutical outcomes based on randomized clinical trials.

## Figures and Tables

**Table 1:** Prevalence of GAVE in patients with systemic sclerosis.

Reference	Cohort characteristics	Prevalence of GAVE	Other variables measured
Ghrénassia et al. [18]	Patients from European League Against Rheumatism Scleroderma Trials and Research. 49 patients were included (24 with diffuse cutaneous SSc).	1%	Diminished DLCO value. Presence of anti-RNA-polymerase III antibodies. Higher association with anemia.

Hung et al. [15]	Patients from Scleroderma: Cyclophosphamide Or Transplant (SCOT) trial. 103 patients diagnosed by endoscopy.	22.3%	No association between anti-RNA polymerase III and GAVE. Presence of vascular ectasia in other parts of the stomach.

Ingraham et al. [19]	Patients from the Division of Rheumatology at Georgetown University and Thomas Jefferson University. 28 patients, 17 with diffuse cutaneous and 11 with limited cutaneous systemic sclerosis.	76%^*∗*^	4% of patients had anti-topoisomerase I antibody.

Marie et al. [14]	264 patients with systemic sclerosis between 1900 and 2008.	5.7%	Systemic sclerosis onset preceded watermelon stomach manifestations in 13 patients (86.7%).

^*∗*^Patients with diffuse cutaneous systemic sclerosis within 18 months of the first symptoms.
